# Pimozide and Adipic Acid: A New Multicomponent Crystalline Entity for Improved Pharmaceutical Behavior

**DOI:** 10.3390/molecules29235610

**Published:** 2024-11-27

**Authors:** Alessandra Buscarini, Michael J. Zaworotko, Catiúcia R. M. O. Matos, Fabrizia Grepioni, Laura Contini, Doretta Capsoni, Valeria Friuli, Lauretta Maggi, Giovanna Bruni

**Affiliations:** 1 Department of Chemistry, Physical Chemistry Section & C.S.G.I. (Consorzio Interuniversitario per lo Sviluppo dei Sistemi a Grande Interfase), University of Pavia, Via Taramelli 16, 27100 Pavia, Italy; alessandra.buscarini01@universitadipavia.it (A.B.); doretta.capsoni@unipv.it (D.C.); 2 Department of Chemical Sciences and Bernal Institute, University of Limerick, V94 T9PX Limerick, Ireland; xtal@ul.ie (M.J.Z.); catiuciarmom@gmail.com (C.R.M.O.M.); 3 Dipartimento di Chimica “Giacomo Ciamician”, Università di Bologna, Via Selmi 2, 40126 Bologna, Italy; fabrizia.grepioni@unibo.it (F.G.); laura.contini3@unibo.it (L.C.); 4 Department of Drug Sciences, University of Pavia, Viale Taramelli 12, 27100 Pavia, Italy; valeria.friuli@unipv.it (V.F.); lauretta.maggi@unipv.it (L.M.)

**Keywords:** pimozide, adipic acid, pharmaceutical salts, Differential Scanning Calorimetry, powder X-ray diffraction, single crystal X-ray diffraction, solubility, biopharmaceutics classification system’s class II

## Abstract

Pimozide is a first-generation antipsychotic used in the treatment of schizophrenia, Gilles de la Tourette syndrome, and other chronic psychoses. Its in vivo efficacy is limited by poor solubility and consequent poor bioavailability. Therefore, adipic acid was used as a coformer for the preparation of a binary product with improved pharmaceutical properties. The thermal behavior of the liquid-assisted grinding products of compositions included in the range 0.1 < *X_PMZ_* < 0.9 has been interpreted using a thermo-dynamic model according to which the two components originate a new crystalline entity in molar ratio pimozide:adipic acid 0.66:0.33, which forms an eutectic system with adipic acid. The model was confirmed using the quantitative analysis of the melting peaks and using the X-ray diffraction measurements from powders and single crystals. In particular, the latter have demonstrated that the new entity resulting from the pimozide:adipic acid 0.66:0.33 composition is actually salt *[PMZH]*_2_*[adipate]*. The crystalline product was characterized, from a pharmaceutical perspective, in terms of solubility and wettability (contact angle). Then, a tablet formulation was developed, and its dissolution behavior was compared to a commercial product considered as a reference. The new entity showed improved pharmaceutical properties in terms of solubility and wettability compared to the pure drug in both deionized water and bio-relevant fluids simulating oral administration in fed and fasted conditions. The tablets containing the new crystalline form can make this virtually insoluble drug available for absorption within minutes regardless of the variability in gastric conditions.

## 1. Introduction

Pimozide (*PMZ*), 1-(1-(4,4-bis(p-fluorophenyl)butyl)-4-piperidyl)-2-benzimidazoline, is a first generation antipsychotic, synthesized in 1963 by Janssen Pharmaceutica [1]. First generation or typical antipsychotic-like pimozide are classified based on their relative affinities for the dopamine D2 receptor, either as low potency (e.g., thioridazine and chlorpromazine) or high potency (e.g., haloperidol). Both the therapeutic and side effects of these drugs rely on the blockade of D2 receptors. 

Traditionally used to treat the “positive symptoms” of schizophrenia, such as hallucinations, delusions, paranoia, and disorganization of thought, pimozide has shown efficacy in the treatment of chronic psychosis [2,3,4] and delusional parasitosis [1,5,6]. Additionally, in 1984, it was FDA-approved in the United States for treating Gilles de la Tourette syndrome. The primary mechanism of Pimozide is the blockade of the D2 dopamine receptor with minor effects on other neurotransmitters. This selective action reduces noradrenergic blockade-associated adverse effects, such as sedation and orthostatic hypotension [6].

Furthermore, Pimozide has been found to block voltage-dependent calcium channels, which is concerning, as it could result in severe side effects when used in higher dosages [6,7]. The predominant side effects often observed with pimozide use are extrapyramidal reactions, notably characterized by Parkinsonian symptoms. These symptoms encompass tremors, bradykinesia, a shuffling gait, and a masked facial expression. However, the prevalence of these adverse effects depends on the dosage administered [6].

Despite its primarily antipsychotic properties, Pimozide remains a very interesting active principle due to its diverse off-label therapeutic indications. For example, research suggests its potential in treating cancer, indicating promising avenues for further investigation [8,9,10,11]. 

Unfortunately, Pimozide falls into BCS’s class II, resulting in poor aqueous solubility, which can impede both oral bioavailability and clinical response to the drug [12]. The bioavailability of Pimozide after oral administration is in fact 50% [6].

Many active principles exhibit undesirable physicochemical properties such as flowability, compressibility, solubility, dissolution rate, permeability, etc., which hinder the development of solid dosage forms [13]. Among these properties, solubility is the most important, in fact it limits the potential bioavailability of molecules that would otherwise have a significant impact on their physiological target [14]. 

For this reason, enhancing it currently stands as one of the primary challenges for the pharmaceutical sector. Over time, various strategies have been developed. Some commonly used methods include use of co-solvents [15,16], micronisation [17], microemulsification [18], formation of salts [19], and cocrystals [20,21,22], and complexation of drugs with cyclodextrins [23].

Research on multicomponent crystalline forms can lead to more effective drugs with fewer side effects. For this reason, the field of crystal engineering has ever greater importance as a new approach to obtain a pharmaceutical product with improved physicochemical and mechanical properties.

In general, salts are the most commonly adopted strategy in the pharmaceutical industry for ionizable active ingredients; in fact, approximately 50% of approvals by the United States Food and Drug Administration (FDA) involve active ingredients in salt form [24]. 

The primary reasons include the ease of synthesis and crystallization, as well as the low cost of starting materials [25]. Salts not only are able to enhance the solubility, but also can beneficially affect the purity, isolation, stability (both chemical and physical), and manufacturability (particularly flow) of the active principle. They also can influence irritancy/toxicity of the dosage form [26].

As not all active ingredients readily ionize, there has been a growing interest in cocrystals in recent years, offering a promising alternative for enhancing the physicochemical properties of pharmaceutical substances. 

Pharmaceutical cocrystals are crystalline materials composed of two or more different molecules, typically the active ingredient and a coformer, within the same crystalline entity [27].

Cocrystals, as novel crystalline forms, exhibit distinct physicochemical and pharmacokinetic properties, often enhanced [28]. For this reason, the number of publications on cocrystals has been steadily increasing, encompassing a wide range of fields such as pharmaceuticals, nutraceuticals, high-energy materials, fertilizers, food products, cosmetics, etc. [29].

The physicochemical characterization of multicomponent crystalline forms is usually conducted using techniques such as Differential Scanning Calorimetry (DSC), Thermogravimetric Analysis (TGA), X-ray Diffraction—from powder (PXRD) or a single crystal (SC-XRD) when available—and Fourier Transform Infrared Spectroscopy (FT-IR). 

Among these techniques, DSC stands out as an excellent tool that can also be used as a screening method for salts or cocrystals. One reason is that new binary crystalline entities can directly form from eutectic melting during the DSC experiment. 

The DSC screening method relies on heat-induced crystallization and the fact that a combination of drug and coformer capable of forming salts and cocrystals exhibits eutectic-type phase diagrams with the formation of a compound with congruent or incongruent melting points. DSC measurements are useful not only as a screening method to investigate the potential formation of new binary crystalline entities among specific components, but also to determine the composition and melting enthalpy of the new crystalline entity. This method is based on constructing a graph similar to the Tammann’s plot, commonly used for the study of eutectic mixtures, but applied here to the melting peak of the new crystalline entity instead of the eutectic peak. 

**Scheme 1:**
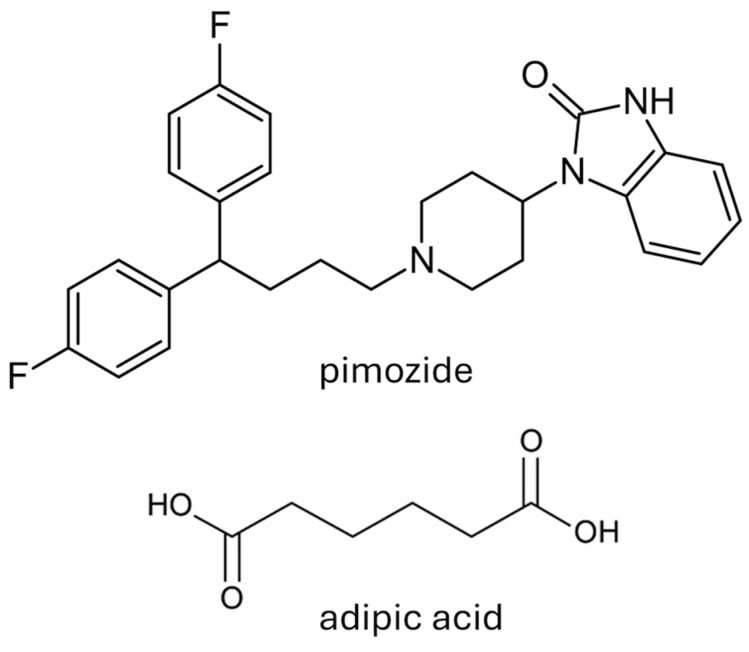
The solid components used in this binary system investigation: pimozide (*PMZ*) and adipic acid (*AAD*).

The aim of the present work is the physico-chemical characterization of a binary system containing pimozide and adipic acid (*AAD*) (see Scheme 1). Adipic acid is a dicarboxylic acid which, at room temperature, appears as a white, odorless crystalline solid. It is included in the GRAS (Generally Recognized as Safe) list; in fact, it can be found as a food additive under the abbreviation E355. The selection of a coformer in the design of pharmaceutical salts or cocrystals is crucial, as it must not only be compatible with the active pharmaceutical ingredient, but also be pharmaceutically acceptable and non-toxic. Among the various anionic and cationic counterions used in FDA-approved pharmaceutical salts, carboxylic acids are among the most common. 

An in-depth thermal study taking advantage of the quantitative potential of DSC was also carried out on the binary systems obtained using liquid assisted grinding (LAG) to gain information on the entire compositional range of this system. 

Single-crystal and powder X-ray diffraction were used for the structural characterization at low- and room temperature for the stoichiometric salt *[PMZH]*_2_*[adipate]*, obtained from the reaction in solution or in the solid state of pimozide and adipic acid in a 2:1 molar ratio.

The crystalline product was characterized from a pharmaceutical point of view in terms of solubility and wettability. Then, a tablet formulation was developed, and its dissolution behavior was compared to pimozide alone or a commercial product considered as a reference in conditions simulating the oral administration.

## 2. Results and Discussion

### 2.1. The Thermal Behavior of Pure PMZ and AAD

**Figure 1:**
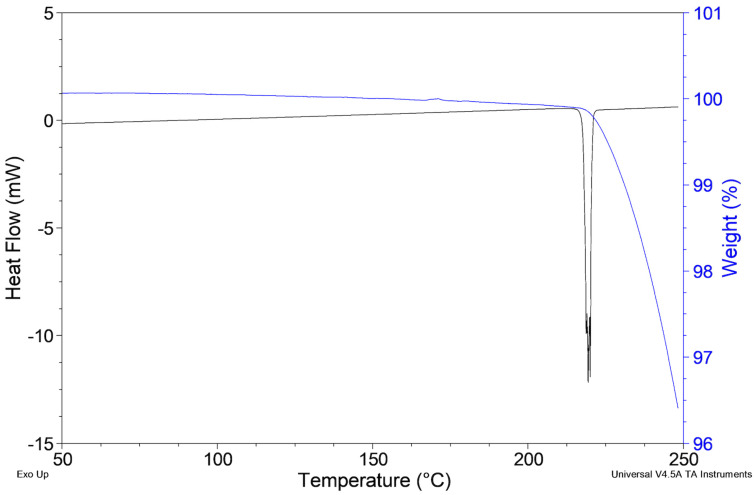
DSC and TGA curves of *PMZ*.

In Figure 1, the DSC and TGA curves of commercial pimozide are shown. A single narrow and indented endothermic peak, typical of a melting event, is present. Its onset temperature (*T*_onset_) is 218.2 ± 0.1 °C, while its enthalpic change (∆*H*) is 107.4 ± 0.6 J g^−1^. A significant mass loss starts from 225 °C, which does not complete even at 260 °C. It probably corresponds to the onset of an evaporation and/or decomposition event following the melting. 

**Figure 2:**
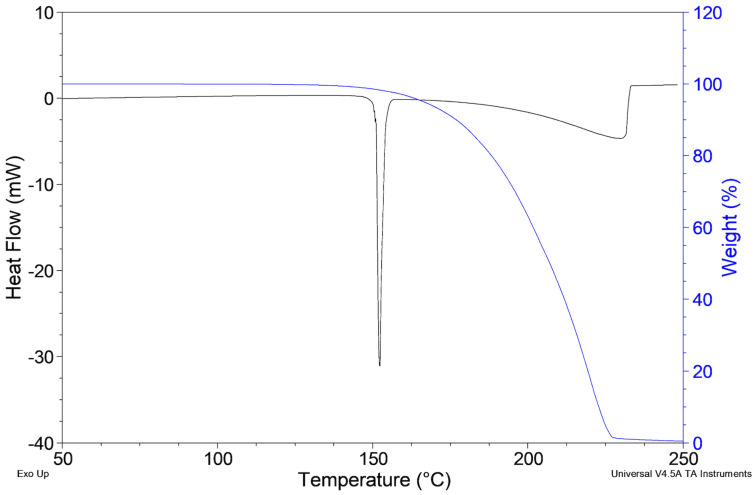
DSC and TGA curves of *AAD*.

The thermal events shown by pure *AAD* are its melting at *T*_onset_ 152.4 ± 0.1 °C (∆*H* = 210.4 ± 0.5 J g^−1^) followed by evaporation of the melt (Figure 2).

### 2.2. Qualitative Analysis of the DSC Curves of the LAG Products with Composition 0.9 ≤ X_PMZ_ ≤ 0.5

The LAG method was used to prepare the binary systems containing amounts of pimozide and adipic acid in different molar ratios. They will be named in the manuscript with an alphanumeric code that clearly indicates the molar ratio of their components. 

The LAG products were analyzed using DSC. Their DSC curves are significantly different from those of the pure components: the peaks typical of the original components are absent and new peaks appear. Thus, a detailed analysis was performed to try to identify the chemical–physical processes at the origin of thermal effects. 

**Figure 3:**
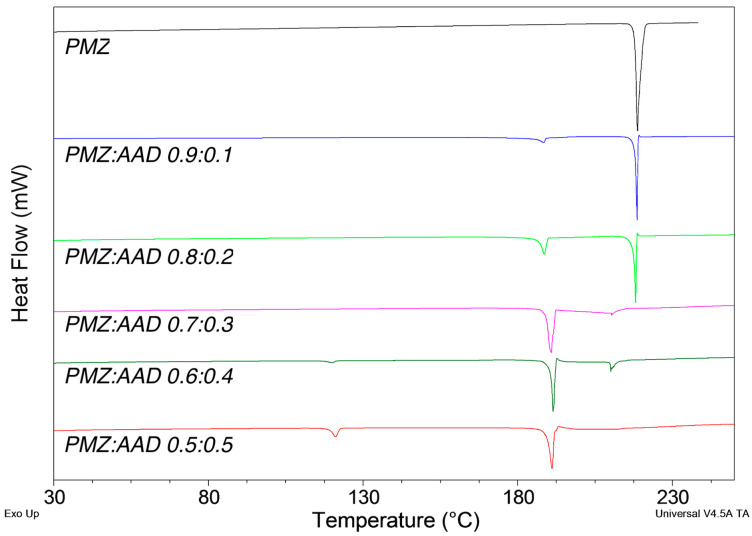
DSC traces of *PMZ* and LAG products with a composition of 0.9 ≤ *X_PMZ_* ≤ 0.5.

In Figure 3, the DSC traces of pure *PMZ* and the samples with varying molar fractions of pimozide ranging from 0.9 to 0.5 are displayed.

The DSC curves of all of these samples show a new endothermic peak associated with a melting process at a temperature between those of the pure components, *T*_onset_ = 188.4 °C. 

This endothermic peak is always present at the same temperature. 

Furthermore, another endothermic effect is observed gradually shifting towards higher temperatures until it coincides with the melting peak of pure *PMZ*.

### 2.3. Qualitative Analysis of the DSC Curves of the LAG Products with Composition 0.4 ≤ X_PMZ_ ≤ 0.1

**Figure 4:**
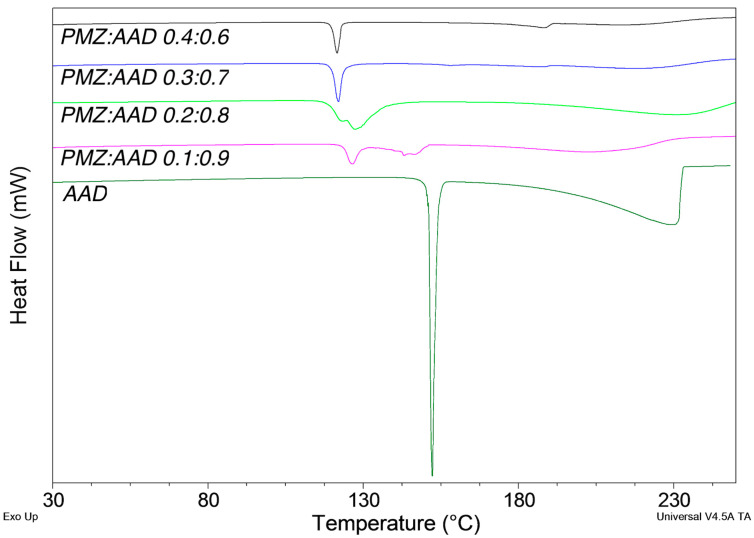
DSC traces of *AAD* and LAG products with a composition of 0.4 ≤ *X_PMZ_* ≤ 0.1.

In Figure 4, the DSC traces of pure adipic acid and LAG products with a molar composition of pimozide 0.4 ≤ *X_PMZ_* ≤ 0.1 are shown.

In the DSC curves of these LAG products a new endothermic peak appears at a constant temperature (*T*_onset_ = 120.6 °C), lower than those of both pure components, while the peak due to *PMZ* melting is absent.

In *PMZ:AAD* 0.4:0.6, a peak at 187.5 °C is present. It is absent in the other compositions.

For the sample *PMZ:AAD* 0.3:0.7, there are no other signals present apart from the peak at 120.6 °C.

In the samples with *X_PMZ_* = 0.2 and 0.1, the peak at 120.6 °C is doubled. 

### 2.4. Thermodinamic Model

The experimental findings suggest that an interaction between the active ingredient and the coformer takes place, leading to the formation of a new crystalline phase that will be denoted as *NC*. It melts at *T*_onset_ = 188.4 °C. Subsequent analyses will ascertain whether *NC* is a cocrystal or a salt. 

In the samples with *X_PMZ_* > 0.5, the second endothermic effect, occurring at variable temperatures, can be attributed to the melting of *PMZ* in excess of the stoichiometric composition of *NC*.

The behavior observed for the samples with *X_PMZ_* < 0.4 agrees with that of a eutectic system. We hypothesize that the components of this eutectic system are pure *AAD* and *NC*. The peak present at the constant temperature of 120.6 °C corresponds to the eutectic melting, while the peak that shifts as the composition varies is due to the melting of the component in excess with respect to the eutectic composition (*PMZ*:*AAD* = 0.3:0.7).

Overall, the two components give rise to an incongruent melting point diagram where the peritectic point *P* falls into composition *PMZ*:*AAD* = 0.4:0.6. 

**Figure 5:**
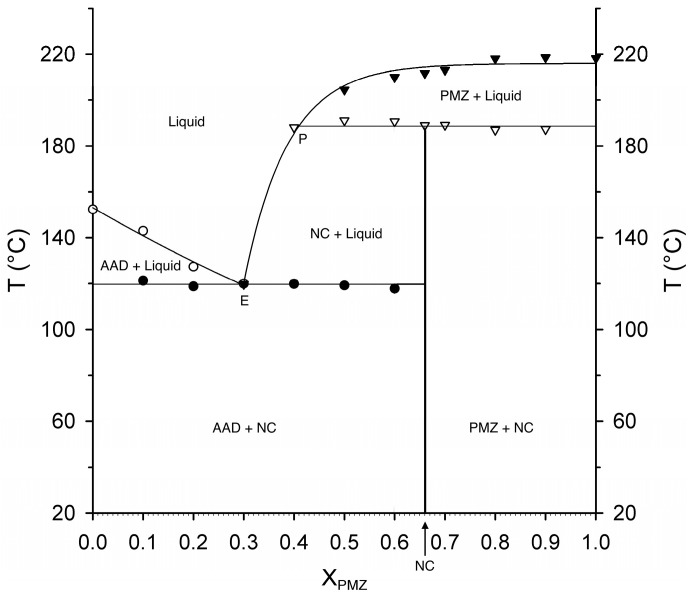
Phase diagram of pimozide and adipic acid.

The phase diagram of our system is shown in Figure 5.

### 2.5. Quantitative Analysis of the DSC Curves

To confirm the validity of the thermodynamic model developed from the qualitative analysis of the DSC curves, a quantitative analysis of the melting enthalpies becomes imperative. 

The DSC instrument provides the specific enthalpy of the thermal effect observed, i.e., the enthalpy per mass unit of the analyzed sample. Thus, when the sample has the same composition of the new binary crystalline entity (whether a salt or a cocrystal), the DSC measurement gives its specific melting enthalpy value. Conversely, the lack of one of the components limits the amount of the binary compound that can form within the sample, and the specific enthalpy measured in the DSC is lower, since only a fraction of the total sample mass is made of the binary compound.

Thus, the measured specific melting enthalpy reaches its maximum value when the sample composition corresponds to that of the binary compound, and gradually reduces until it cancels itself when the relative quantity of one or the other component of the binary mixture approaches zero.

The experimental-specific enthalpy measured for the melting peak of the new binary compound is the product of the specific melting enthalpy of such a compound and its mass fraction in the sample:

(1) $$∆H_{exp}=∆H_{NC}\cdot m_{NC}^{\%}$$

where:

$∆H_{exp}$ = experimental-specific melting enthalpy (J g^−1^);
$∆H_{NC}$ = specific melting enthalpy of the new binary compound (J g^−1^);
$m_{NC}^{\%}$ = mass percentage of the new binary compound in the sample.

If we plot the specific melting enthalpy measured for *NC* melting (*T*_onset_ = 188.4 °C) against the nominal composition (expressed as mass percentage of *PMZ*) of the LAG samples, as it is performed in a Tammann’s plot, two interpolating lines can be drawn (Figure S1). The intersection point of the lines gives the composition and the specific melting enthalpy of *NC*. We found that the composition is *PMZ*:*AAD* = 0.66:0.33 (mol:mol) and its melting enthalpy is 107.7 J/g.

#### 2.5.1. Quantitative Analysis for LAG Samples with Composition 0.9 ≤ *X_PMZ_* < 0.66

With *PMZ:AAD* ratios within the range of 0.9 ≤ *X_PMZ_* < 0.66, the entire amount of *AAD* forms *NC*, leaving an excess of *PMZ*, as evidenced by the DSC curve displaying both the melting peak of *NC* and that of *PMZ*.

In the following, the calculations to determine the expected value of the enthalpy for the melting peak of the new crystalline entity and that of *PMZ* (denoted as ∆*H*_Th,*NC*_ and ∆*H*_Th,*PMZ*_, respectively) are shown. The expected values derived from these calculations are compared with the corresponding experimental values (denoted as ∆*H*_exp,*NC*_ and ∆*H*_exp,*PMZ*_, respectively).

For these calculations, the previously determined value of 107.7 J g^−1^ is used for the *NC* compound-melting enthalpy (∆*H_NC_*).

As long as the amount of *AAD* does not reach the composition of *NC*, *AAD* acts as the limiting component (in respect to the composition of *NC*), and its amount will limit the formation of *NC* within the sample. Thus, for the compositions with 0.9 *≤ X_PMZ_* < 0.66, *AAD* will be the limiting component. The amount of *NC* formed is given by the entire amount of *AAD* and the mass of *PMZ*, which, in agreement with the *NC* composition, will combine with *AAD* in the binary product.

(2) $$m_{PMZ,NC}^{\%}=m_{AAD,NC}^{\%}\cdot \frac{x_{PMZ,NC}}{x_{AAD,NC}}\cdot \frac{{MW}_{PMZ}}{{MW}_{AAD}}$$

(3) $$m_{NC}^{\%}=m_{AAD,NC}^{\%}+m_{PMZ,NC}^{\%}$$

(4) $$∆H_{Th,\;NC}=∆H_{NC}\cdot m_{NC\;\;}^{\%}$$

where:

$m_{NC}^{\%}$ = mass percentage of the new binary compound in the sample;
$m_{AAD,NC}^{\%}$ = mass percentage of adipic acid in *NC*;
$m_{PMZ,NC}^{\%}$ = mass percentage of pimozide in *NC*;
*MW* = molar weight.

According to our model, as a consequence of *NC* incongruent melting, a liquid of composition *PMZ*:*AAD* = 0.4:0.6 (the peritectic composition *P*) and solid *PMZ* are obtained.

Thus, in the DSC curve of these compositions, an endothermic peak due to melting of *PMZ* is present too. Its enthalpic value ($∆H_{Th,\;PMZ}$) can be determined from the amount of *PMZ* in excess respect to the peritectic composition and corresponds to:

(5) $$m_{PMZ,eccP}^{\%}=m_{PMZ}^{\%}-m_{PMZ,P}^{\%}$$

where:

$m_{PMZ,eccP}^{\%}$ = mass percentage of pimozide in excess respect to the peritectic composition;
$m_{PMZ}^{\%}$ = mass percentage of pimozide in the sample;
$m_{PMZ,P}^{\%}$ = mass percentage of pimozide in the peritectic composition.

The amount of $m_{PMZ,P}^{\%}$ can be determined considering that all the amount of *AAD* present in the sample is in the liquid of peritectic composition.

(6) $$m_{PMZ,P}^{\%}=m_{AAD,NC}^{\%}\cdot \frac{x_{PMZ,P}}{x_{AAD,P}}\cdot \frac{{MW}_{PMZ}}{{MW}_{AAD}}$$

(7) $$∆H_{Th,\;PMZ}=∆H_{PMZ}\cdot m_{PMZ,eccP}^{\%}$$

In Table 1, the experimental enthalpy values and those expected for the melting of the new crystalline entity *NC* and of the excess *PMZ* present in samples of the various compositions are shown.

It is evident that the expected and the experimental values of the *NC* melting enthalpy are in excellent agreement, proving our model. 

#### 2.5.2. Quantitative Analysis for LAG Samples with Composition 0.4 < *X_PMZ_* ≤ 0.1

According to our model, for compositions within this range, the relative amount of *AAD* increases beyond the value of the composition of the new binary compound. Consequently, the amount of *NC* that can be formed will be limited by the component in defect, i.e., *PMZ*.

The qualitative analysis of the DSC curves suggests that for compositions with 0.4 *< X_PMZ_ ≤* 0.1:

- The entire amount of *PMZ* participates in the *NC* formation, and *AAD* is in excess;
- *NC* and *AAD* form a eutectic mixture (coded as *EU*) with a composition of *X_PMZ_* = 0.3.

##### Quantitative Analysis for LAG Sample with Composition *X_PMZ_* = 0.3

The nominal composition of this sample coincides with that of *EU*. 

Let us define the following: $m_{AAD,ecc}^{\%}$ = mass percentage of adipic acid in excess respect to *NC*.

(8) $$m_{AAD,NC}^{\%}=m_{PMZ,NC}^{\%}\cdot \frac{x_{AAD,NC}}{x_{PMZ,NC}}\cdot \frac{{MW}_{AAD}}{{MW}_{PMZ}}$$

(9) $$m_{NC}^{\%}=m_{AAD,NC}^{\%}+m_{PMZ,NC}^{\%}$$

(10) $$m_{AAD,ecc}^{\%}=m_{AAD}^{\%}-m_{AAD,NC}^{\%}$$

*NC* and *AAD* in this sample are stoichiometric in respect to *EU*; therefore, the entire amounts of *NC* and *AAD* form *EU*.

(11) $$m_{EU}^{\%}=m_{NC}^{\%}+m_{AAD,ecc}^{\%}=100\%$$

It can be easily derived that the molar composition of *EU* is *X_NC_* = 0.20 *X_AAD_* = 0.80. 

The enthalpy change measured for the peak at 120.0 °C in the curve of this sample is the specific melting enthalpy of the eutectic mixture: $∆H_{EU\;}$= 88.55 ± 2.7 J g^−1^.

##### Quantitative Analysis for LAG Samples with Composition *X_PMZ_* = 0.2 and 0.1

The samples *PMZ:AAD* 0.2:0.8 and *PMZ:AAD* 0.1:0.9 are made of *EU* (*NC* + *AAD*) and *AAD* in excess respect to the *EU* composition. In the DSC curve, the eutectic melting peak and the excess *AAD* melting peak are not separated, and only the sum of their enthalpies can be measured ($∆H_{\;exp,TOT})$. The expected and the experimental values obtained for these compositions are reported in Table 2. The expected values are derived as follows.

Let us define the following: $m_{AAD,eccEU}^{\%}\;$ = mass percentage of *AAD* in excess respect to *EU*.

(12) $$m_{AAD,NC}^{\%}=m_{PMZ,NC}^{\%}\cdot \frac{x_{AAD,NC}}{x_{PMZ,NC}}\cdot \frac{{MW}_{AAD}}{{MW}_{PMZ}}$$

(13) $$m_{NC}^{\%}=m_{PMZ,NC}^{\%}+m_{AAD,NC}^{\%}$$

(14) $$m_{AAD,\;ecc}^{\%}=m_{AAD}^{\%}-m_{AAD,NC}^{\%}$$

(15) $$m_{AAD,\;EU}^{\%}=m_{NC}^{\%}\cdot \frac{x_{AAD,EU}}{x_{NC,EU}}\cdot \frac{{MW}_{AAD}}{{MW}_{NC}}$$

(16) $$m_{EU}^{\%}=m_{NC}^{\%}+m_{AAD,\;EU}^{\%}$$

(17) $$m_{AAD,\;eccEU}^{\%}=m_{AAD,\;ecc}^{\%}-m_{AAD,\;EU}^{\%}$$

(18) $$∆H_{Th,\;EU}=∆H_{EU}\cdot m_{EU}^{\%}$$

(19) $$∆H_{Th,\;AAD}=∆H_{AAD}\cdot m_{AAD,\;eccEU}^{\%}$$

As can be observed, considering the total integration of the peaks due to the melting of the eutectic mixture and excess *AAD*, there is good agreement between the expected and experimental values.

##### Quantitative Analysis for LAG Sample with Composition 0.4 ≤ *X_PMZ_* ≤ 0.6

These samples are made of *EU* (*NC* + *AAD*) and *NC* in excess respect to the *EU* composition. Indeed, in their DSC curves, both the eutectic melting peak and the excess *NC* peak are present. The sample *PMZ:AAD* 0.4:0.6 has the peritectc composition.

Let us define the following: $m_{NC,eccEU}^{\%}$ = mass percentage of *NC* in excess respect to *EU.*

(20) $$m_{AAD,\;ecc}^{\%}=m_{AAD}^{\%}-m_{AAD,\;NC}^{\%}$$

(21) $$m_{NC,\;EU}^{\%}=m_{AAD,\;ecc}^{\%}\cdot \frac{x_{NC,EU}}{x_{AAD,EU}}\cdot \frac{{MW}_{NC}}{{MW}_{AAD}}$$

(22) $$m_{EU}^{\%}=m_{NC,\;EU}^{\%}+m_{AAD,\;ecc}^{\%}$$

(23) $$m_{NC,\;eccEU}^{\%}=m_{NC}^{\%}-m_{NC,\;EU}^{\%}$$

(24) $$∆H_{Th,\;EU}=∆H_{EU}\cdot m_{EU}^{\%}\;$$

(25) $$∆H_{Th,\;NCeccEU}=∆H_{NC}\cdot m_{NC,\;eccEU}^{\%}$$

According to our model, as a consequence of *NC* incongruent melting, a liquid with the composition *PMZ*:*AAD* = 0.4:0.6 (the peritectic composition) and solid *PMZ* are obtained.

Thus, in the DSC curves of these compositions an endothermic peak due to melting of *PMZ* is present too. The data processing to calculate its expected enthalpy change is similar to that already exposed for the compositions 0.7 < *X_PMZ_* < 0.9.

In Table 3, the experimental enthalpy values and those expected for the melting of *EU*, NC in excess respect to *EU*, and of the excess *PMZ* present in samples of the various compositions are reported. 

It is evident that the expected and the experimental values of the *NC* melting enthalpy are in agreement, proving our model. 

##### Quantitative Analysis for LAG Sample with Composition *X_PMZ_* = 0.66

A sample *PMZ:AAD* 0.66:0.33 was also prepared using the LAG method. It is made of pure *NC*. Indeed, the experimental enthalpy change measured for *NC* melting perfectly agrees with that expected for pure *NC*.

In the DSC curve, the *PMZ* peak is present too. Thus, from the melting of an *NC*, a liquid with peritectic composition and solid *PMZ* are expected.

The total amount of *AAD* will be present in the peritectic melt.

(26) $$m_{PMZ,P}^{\%}=m_{AAD,\;P}^{\%}\cdot \frac{x_{NC,P}}{x_{AAD,P}}\cdot \frac{{MW}_{NC}}{{MW}_{AAD}}$$

(27) $$m_{PMZ,\;eccP}^{\%}=m_{PMZ}^{\%}+m_{PMZ,P}^{\%}$$

(28) $$∆H_{Th,PMZ}=∆H_{PMZ}\cdot m_{PMZ,eccP}^{\%}=61.8\;J\;g^{-1}\;$$

(29) $$∆H_{exp,\;PMZ}=76.4\pm 14.4\;J\;g^{-1}\;$$

### 2.6. TGA of the LAG Products

The mass of the *PMZ:AAD* 0.66:0.33 sample remains constant until approximately 200 °C (Figure S2), a temperature higher than the closure temperature of the DSC peak attributed to melting. Subsequently, the sample begins to lose mass significantly, presumably due to the product decomposition.

### 2.7. Powder X-Ray Diffraction Analysis

**Figure 6:**
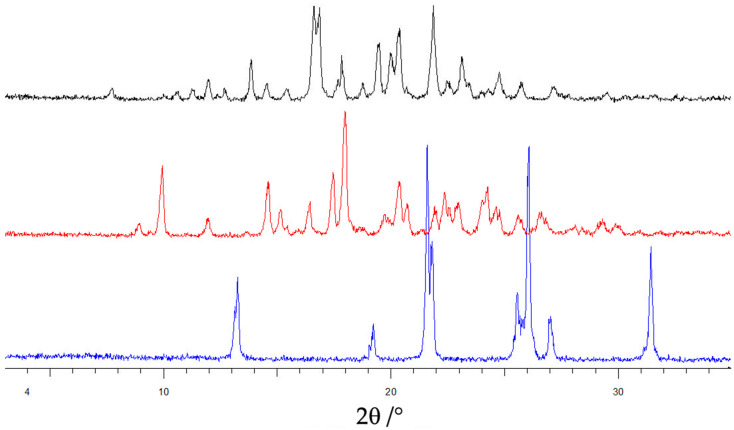
PXRD patterns of pimozide (black), *PMZ:AAD* 0.66:0.33 (red), and adipic acid (blue).

In Figure 6, the patterns of pure *PMZ* and *AAD*, as well as the LAG products *PMZ:AAD* 0.66:0.33, are shown. 

The patterns of the pure components show well-defined peaks which highlight their crystalline nature. 

The diffraction pattern of the *PMZ:AAD* 0.66:0.33 sample is significantly different from those of the pure components for the absence of their most intense peaks and for the appearance of new ones. In addition to this, Figures S3–S6 report the comparisons between *PMZ*:*AAD* compositions 0.7:0.3, 0.5:0.5, and 0.3:0.7 and the PXRD patterns for the reagents and the 0.66:0.33 calculated pattern, showing that, for all of the compositions different from 0.66:0.33, peaks are invariably observed, indicating the presence of unreacted pimozide or adipic acid. These observations provide further confirmation for the validity of our model.

### 2.8. FT-IR Analysis

**Figure 7:**
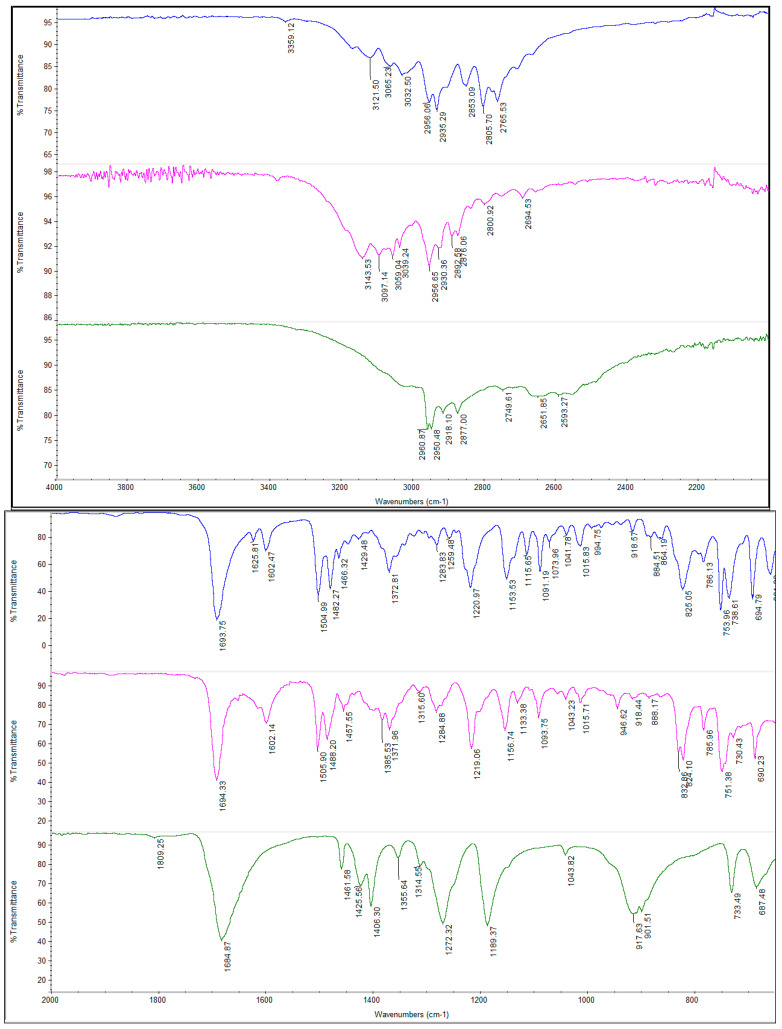
FT-IR spectra of *PMZ* (blue), *PMZ:AAD* 0.66:0.33 (fuchsia), and *AAD* (green).

In Figure 7, the FT-IR spectra of the coformer and the active ingredient are compared with that of *PMZ:AAD* 0.66:0.33. In Table 4 and Table 5, the assignments of peaks and bands present in the spectra of pure *PMZ* and *AAD* to the vibrational modes of the pimozide and *AAD* molecules are listed.

The spectrum of the binary product shows significant differences compared to those of the pure components. The differences mainly concern the absorptions of the NH and OH groups and C-H bonds, as well as those of the amide function and C-F bonds (see peaks and bands at 3143.53 cm^−1^, 2930.36 cm^−1^, 2892.58 cm^−1^, 2800.92 cm^−1^, 1488.20 cm^−1^, 1156.74 cm^−1^, 1133.38 cm^−1^, and 832.86 cm^−1^ present in the spectrum of *PMZ:AAD* 0.66:0.33). They agree with the formation of a hydrogen bonding network involving the active ingredient and the coformer, confirming the conclusions drawn from the mentioned measurements that a new solid phase has formed in the *PMZ:AAD* 0.66:0.33 sample. Furthermore, the new peaks at 1616.14 cm^−1^ and 1385.53 cm^−1^ could be due to the absorpion of a carboxylate group, and this might indicate the formation of a salt.

### 2.9. SEM-EDS Analysis

**Figure 8:**
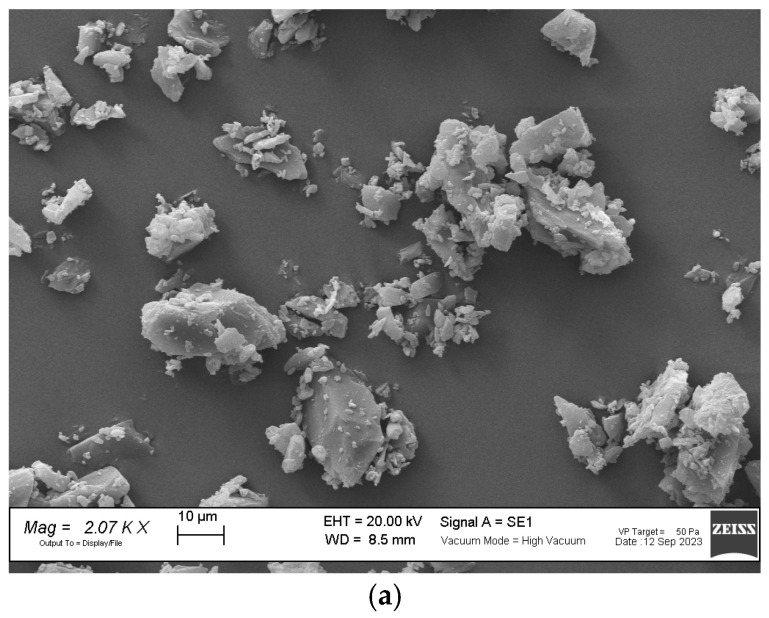

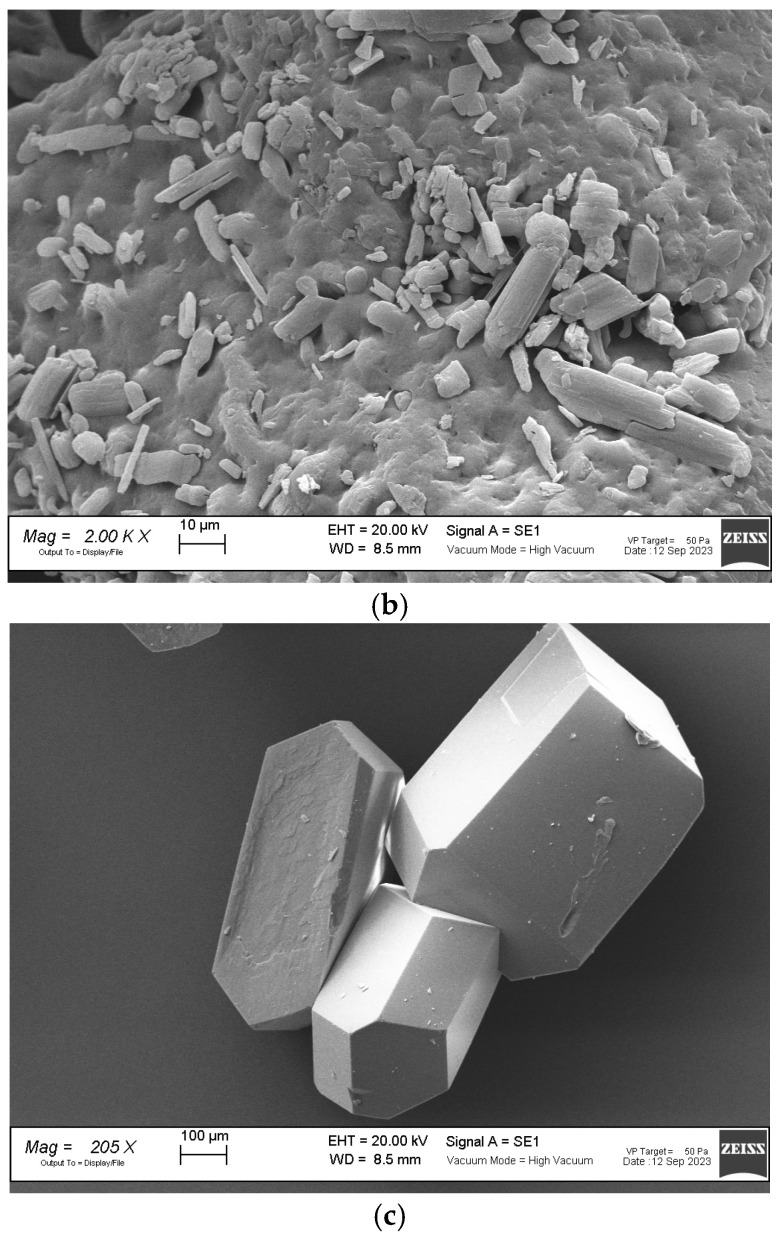
SEM photographs of pimozide (**a**), adipic acid (**b**), and *PMZ:AAD 0.66:0.33ev* (**c**).

The powder of *PMZ* (Figure 8a) is made of particles with an undefined shape and size ranging from some hundreds of nanometers to a few tens of micrometers. They are often stuck together.

The adipic acid appears as irregularly shaped blocks hundreds of microns large and with a non-smooth surface on which small rods can be observed (Figure 8b). 

The *PMZ:AAD 0.66:0.33ev* sample (Figure 8c) is made of beautiful big crystals with clear and defined edges.

**Figure 9:**
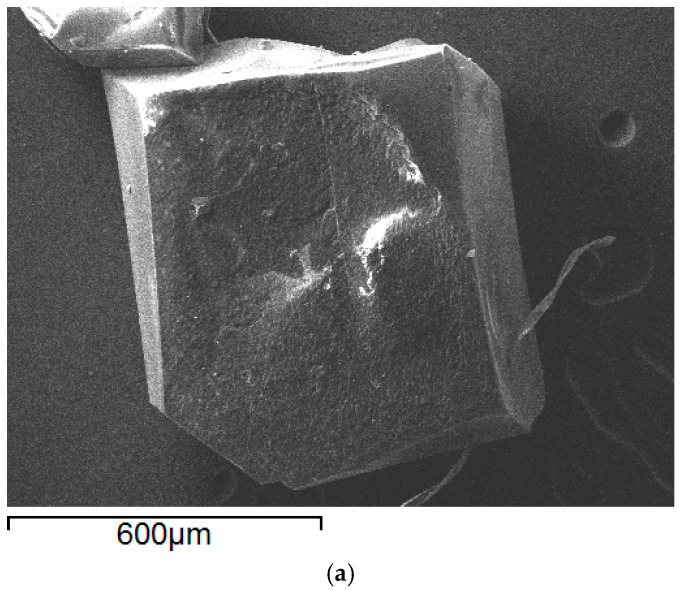

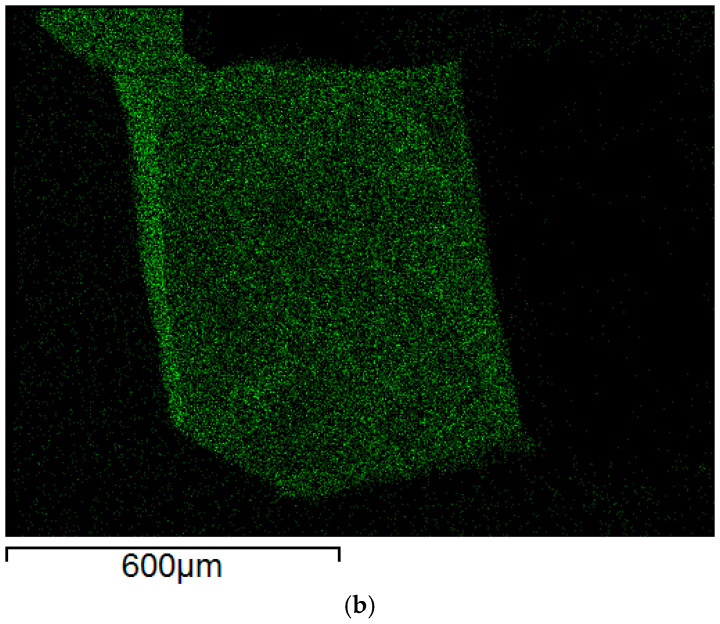
SEM photograph (**a**) and map of Fluorine (**b**) for *PMZ:AAD 0.66:0.33ev*.

The coupled SEM-EDS technique was used to confirm the formation of the multicomponent system. Thus, the map of fluorine, which is present only in the drug molecule, was recorded for the samples of *PMZ:AAD 0.66:0.33ev* (Figure 9). As is evident comparing Figure 9a,b, the fluorine uniformly distributed in the particles and the map (Figure 9b) accurately reproduces the sample morphology (Figure 9a). This again confirms that the sample is not a simple mixture of the two components, but that an interaction between the components, leading to the formation of multicomponent crystals, has occurred.

### 2.10. SC-XRD Measurements

**Figure 10:**
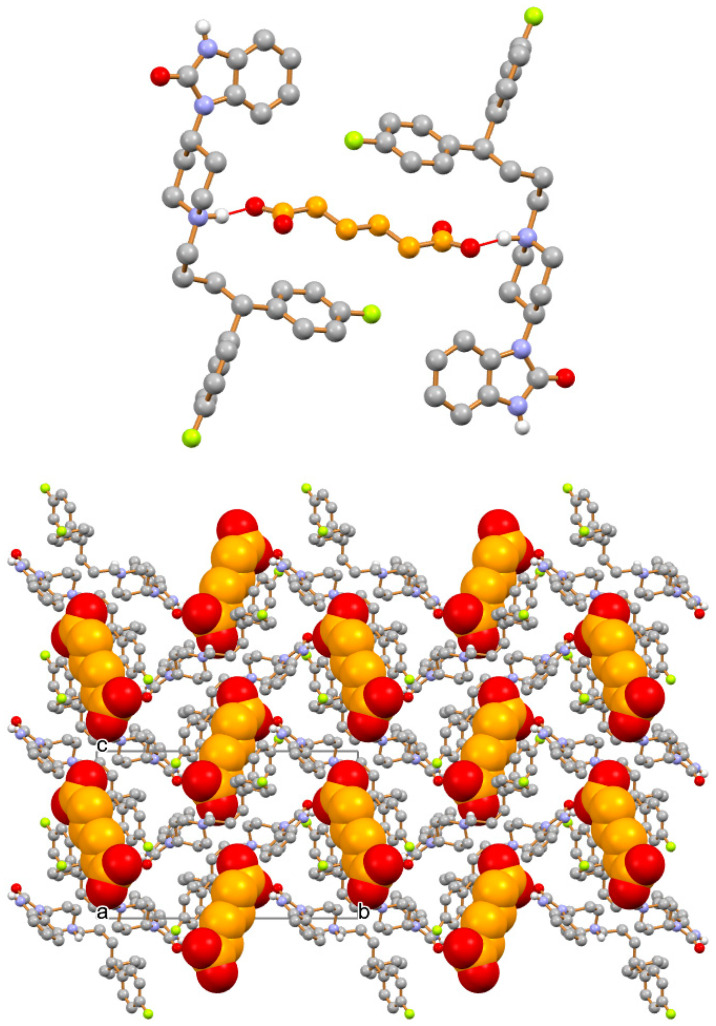
(**Top**) The formula unit of the *[PMZH]*_2_*[adipate]* salt at room temperature. (**Bottom**) Each adipate dianion interacts via charge-assisted hydrogen bonds with four [*PMZH*]^+^ cations, giving rise to an extended 3D-HB network. [Adipate C atoms in orange; H_CH_ atoms omitted for clarity].

Single-crystals X-ray diffraction data were collected on crystalline material obtained from a solution crystallization of pimozide:adipic acid in a 2:1 molar ratio. Data were collected at 150 K and at room temperature. At both temperatures, the compound behaves as a salt, clearly showing proton transfer between the two carboxylic groups on the adipic acid and the piperidinyl nitrogen atom on pimozide (see Figure S7). The pimozide cation occupies a general position in the unit cell, while the adipate dianion lies on a center of inversion, in agreement with a cation:anion 2:1 stoichiometric ratio (Figure 10, top). Each adipate dianion interacts via hydrogen bonds with four [*PMZH*]^+^ cations, giving rise to an extended 3D-HB network that imparts the directionality to the ionic components, being in turn reinforced by the ionic charges present on the -NH^+^ and -COO^−^ groups of the *PMZH*^+^ cation and the adipate dianion, respectively (Figure 10, bottom).

### 2.11. Solubility

The solubility of *PMZ:AAD 0.66:0.33ev* is much improved compared to pure *PMZ* (Table 6). Its value doubles in the fluid at pH = 2, increases more than four times at pH = 4.5, and about six times in deionized water.

### 2.12. CONTACT Angle

**Figure 11:**
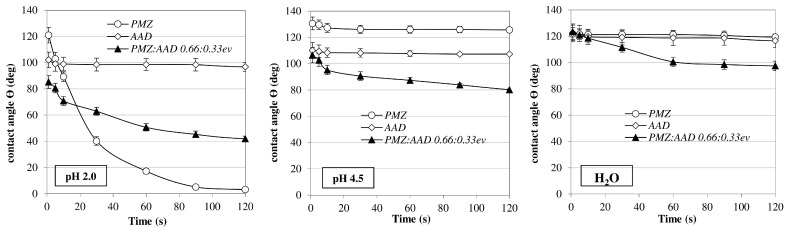
Contact angle, θ, of *PMZ:AAD 0.66:0.33ev* compared to *PMZ* and *AAD* alone in the different fluids considered.

At pH 2, *PMZ* is more soluble and thus wettable: the contact angle changes from 120 θ to 4 θ in about 3 min. In these conditions, *AAD* is a lipophilic molecule and poorly wettable, but the formation of the crystalline product shows an improvement in wettability (Figure 11). Completely different results are obtained in the other two fluids in which the drug is practically insoluble. It is also completely hydrophobic (about 130 θ at pH 4.5 and 120 θ in water). However, the formation of the crystalline product increases the wettability of the compound in comparison to *AAD*. In fact, the contact angle of *PMZ:AAD 0.66:0.33ev* is less than 90 θ at pH 4.5 and less than 100 θ in deionized water: this increase in wettability could significantly accelerate the passage of the drug into the solution.

### 2.13. Dissolution Tests

**Figure 12:**
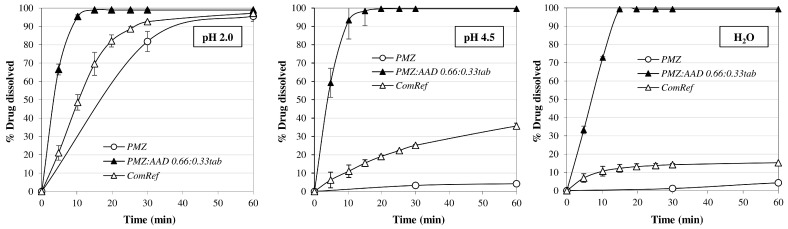
Dissolution profiles of *PMZ* and *PMZ:AAD 0.66:0.33tab* in the three fluids considered compared to the reference commercial product in tablets *ComRef*. All samples contain the same dose of 4 mg of drug.

These improvements in solubility and wettability of the new compound suggest that the biopharmaceutical properties of *PMZ:AAD 0.66:0.33ev* could also be improved following oral administration. A tablet formulation was then developed to evaluate the dissolution behavior of the new crystalline entity compared to a commercial reference. In vitro dissolution tests were then conducted in fluids simulating the conditions of the upper gastrointestinal tract. The results obtained from the tablets containing the new compound (*PMZ:AAD 0.66:0.33tab*) are shown in comparison with pure pimozide and the commercial tablet form (*ComRef*) in the different fluids considered in Figure 12. At pH 2, there showed a condition that simulates administration on an empty stomach and the fluid required by the monograph of the American Pharmacopoeia [Pimozide Tablets, Official Monograph]. In The United States Pharmacopeia (USP43-NF38) [United States Pharmacopeia Convention, Inc.: Rockville, MD, USA, 2023], the drug is more soluble, and in fact all of the samples can release the dose of the drug within an hour. However, only the formulation *PMZ:AAD 0.66:0.33tab* guarantees a prompt release for absorption in about 10 min. The major differences can be noted in the other two fluids: pH 4.5, which simulates an administration on a fed stomach, and deionized water, a neutral condition both in chemical and pH terms. At pH 4.5, only the tablets containing the new compound *PMZ:AAD 0.66:0.33tab* release the drug in a very short time, while just a very tiny fraction of the dose of pure pimozide passes into the solution in the first hour and only half of the dose is released from the commercial product in the same time. As the pH increases, the solubility decreases further; in fact, in neutral conditions (i.e., deionized water), the passage into solution is further depressed for both pure pimozide and the commercial form, while the tablets containing the new crystalline form are still able to guarantee the dissolution of the drug dose within 15 min. The result is making the passage of the drug into the solution much more independent of the pH conditions, at least in the range attributable to the fluids of the upper gastrointestinal tract.

## 3. Materials and Methods

### 3.1. Materials

Commercially available *PMZ* (purity 99.9%) and *AAD* (purity 99%) were purchased from Sigma Aldrich (Milan, Italy) and used as received. The solvents ethanol and DMSO were purchased from Sigma Aldrich (Milan, Italy) and used without further purification. Microcrystalline cellulose NF (Avicel^®^ PH101) was supplied by FMC BioPolymer (Brussels, Belgium), sodium starch glycolate (Explotab) by Mendell (Reigate, Surrey, UK), polyoxyethylene polyoxypropylene triblock copolymer, Poloxamer 407, (Lutrol F127 MICRO) was supplied by BASF (Ludwigshafen, Germany), sodium lauryl sulfate and magnesium stearate by Carlo Erba (Milan, Italy), and silicon dioxide Syloid 72FP was supplied by Grace Davison (Worms, Germany). All other reagents are of analytical grade.

A commercial product in a tablet (*ComRef*) containing 4 mg of *PMZ* (Lusomedicamenta Sociedade Tecnica Farmaceutica S.A., Barcarena, Brazil) was used as a reference.

### 3.2. LAG Method 

*PMZ* and *AAD* in various molar ratios (about 300 mg) were manually ground using an agate mortar and pestle for about 40 min, with the progressive addition of 2 or 3 drops of DMSO. The products were then left to dry under ambient conditions for a week and lightly ground to break the solid crust obtained.

### 3.3. Preparation of Single Crystals 

Amounts of *PMZ* and *AAD* corresponding to the molar ratio 0.66:0.33 were dissolved in a mixture of ethanol and DMSO (1:1 *v*:*v*) heated to about 60 °C under moderate magnetic stirring at 260 rpm for 15 min. The solution was then filtered and left for evaporation until crystals of *PMZ:AAD 0.66:0.33ev* with suitable size for single-crystal X-ray diffraction (SC-XRD, Bruker, Billerica, MA, USA) experiments were obtained.

### 3.4. Physical Mixture

A physical mixture of the two components, *PMZ:AAD 0.66:0.33pm*, was prepared in the 0.66:0.33 molar ratio by mixing the products in a Turbula mixer for 10 min.

### 3.5. Differential Scanning Calorimetry

Thermal analysis of the samples was conducted using a DSC Q2000 instrument interfaced with a TA 5000 data station (TA Instruments, New Castle, DE, USA). The DSC calibration was performed using ultrapure indium (99.999%; melting point = 156.6 °C; ∆*H* = 28.54 J g^−1^) as standard. Samples weighing between 3.5 and 4 mg were scanned at a heating rate of 2 K min^−1^ under a nitrogen flow (45 mL min^−1^) in open aluminum pans. All of the experimental thermal data are averages of at least three experiments. Typically, temperatures were reproduced within 1 °C.

### 3.6. Thermogravimetric Analysis

Thermogravimetric measurements were conducted using a TGA Q5000 analyser connected to a data acquisition and processing system TA5000 (TA Instruments, New Castle, DE, USA). The thermogravimetric measurements were performed on a powder amount ranging from 3 to 4 mg under a nitrogen flow (45 mL min^−1^) at a heating rate of 2 K min^−1^. All experimental thermal data are averages of at least three experiments. Typically, temperatures were reproduced within 1 °C.

### 3.7. Powder X-Ray Diffraction

The powder diffraction patterns were acquired using a Bruker D5005 diffractometer (Siemens, Karlsruhe, Germany), equipped with a vertical goniometer, a curved graphite monochromator, and a scintillation detector. Cu Kα radiation (λ = 1.54056 Å) was utilized. Measurements were conducted at room temperature over the angular range 2θ from 5 to 30° (voltage = 40 kV, current intensity = 40 mA) in step-scanning mode (step size = 0.020°; counting time = 3 s per step). 

### 3.8. Single-Crystal X-Ray Diffraction

Single-crystal X-ray diffraction data at 150 K was collected on a D8 Quest diffractometer equipped with a IμS micro-focus Mo anode Mo Kα (λ = 0.71 Å) and Photon II detector. For low-temperature measurements, an open-flow nitrogen attachment from Oxford Cryosystems was used. The data were indexed in APEX4 (v2021.10-0). Integration was performed using SAINT V8.40A in APEX4.v. Absorption correction was performed using a multi-scan method using SADABS in APEX4.2. Space group determinations were performed with the assistance of XPREP, as implemented in APEX4. Structures were solved using the intrinsic phasing method (SHELXT) and refined on F2 using SHELXL least squares method [30], as run in OLEX2 v1.3 program packages [31]. All non-hydrogen atoms on the frameworks were refined anisotropically. Hydrogen atoms were added geometrically at idealized positions and refined using the riding model.

Single-crystal X-ray diffraction data at room temperature were collected with an Oxford Diffraction X’Calibur equipped with a graphite monochromator and a CCD detector (Oxford Instruments, Oxford, UK). Mo−Kα radiation (λ = 0.71073 Å) was used. Structural solution and refinement using least-squares methods against F^2^ were carried out using SHELXT and SHELXL [30] implemented in the Olex2 [31] software. Non-hydrogen atoms were refined anisotropically. Highest peaks of residual electron density from Fourier maps indicated the position of the hydrogen atom belonging to -NH group on the pimozide cation, thus confirming the salt attribution. H_CH_ hydrogens were added in calculated positions and refined riding on their respective N or O atoms. The program Mercury [32] was used for graphic representations. Both the RT and LT pimozide cations show the analogous positional disorder (80:20 and 75:25, respectively) of the aliphatic -CH_2_-CH_2_-CH_2_- fragment. 

Crystal data and details of measurements at RT and 150 K are listed in Table S1. CCDC 2389843–2389844 contains the supplementary crystallographic data for this paper. These data can be obtained free of charge via www.ccdc.cam.ac.uk/data_request/cif, or by emailing data_request@ccdc.cam.ac.uk, or by contacting The Cambridge Crystallographic Data Centre, 12 Union Road, Cambridge CB2 1EZ, UK; Fax: +44-1223-336033.

### 3.9. Fourier Transform Infrared Spectroscopy

FT-IR measurements were performed using an iS10 FT-IR spectrophotometer (Nicolet, Madison, WI, USA) equipped with an ATR (Attenuated Total Reflectance) sampling accessory (Smart iTR model with a diamond crystal). Thirty-two scans were averaged over the wavenumber range of 4000–550 cm^−1^ with a resolution of 4 cm^−1^.

### 3.10. Scanning Electron Microscopy Coupled with Energy Dispersive X-Ray Analysis

A Zeiss EVO MA10 (Carl Zeiss, Oberkochen, Germany) scanning electron microscope coupled with an EDS detector (X-max 50 mm^2^, Oxford Instruments, Oxford, UK) was used for the morphologic study (on previously gold sputtered samples) and the elemental microanalysis of the samples. 

### 3.11. Solubility

The samples were sieved through a 230-mesh grid, 63 μm (Endecotts, London, UK).

The spectra of *AAD* and *PMZ* were recorded previously to prevent the possibility of interference in the absorbance of the two molecules. The solubility of *PMZ* and *PMZ:AAD 0.66:0.33ev* were determined by the shake flask method with spectrophotometric determination (Lambda 25, Perkin-Elmer, Monza, Italy) in deionized water, 0.01N hydrochloric acid pH = 2, and phosphate buffer pH = 4.5. An excess sample is placed in the flasks containing the three different fluids and left under stirring. At time intervals, a portion of the solution was taken and filtered on a 0.22 μm filter (Millipore Merck, Darmstadt, Germany). The drug concentration was determined using UV detection (Lambda 25, Perkin-Elmer, Monza, Italy). The test was repeated until equilibrium was reached. The spectra of *AAD* and *PMZ* were recorded previously to prevent the possibility of interference in the absorbance of the two molecules.

### 3.12. Contact Angle

In total, 120 mg of each sample was placed in a cylindrical ring, and the surface was smoothed with a scraper. The Contact Angle Meter DMe-211Plus (NTG Nuova Tecnogalenica, Cernusco, Italy) was used to record the images of a drop (9 μL) of the different fluids once placed in contact with the surface of the samples at progressive times. The contact angles were measured using the software supplied with the apparatus (three replicates).

### 3.13. Tablet Formulation

Then, a tablet formulation was designed with the composition reported in Table 7. *PMZ:AAD 0.66:0.33ev* and the excipients were mixed in a Turbula apparatus (Willy Bachofen, Basel, Switzerland) for 15 min. The blend was then compressed using a single-punch tablet machine (Korsch EK0; Korsch AG, Berlin, Germany) equipped with 8 mm in diameter flat punches at 25 kN.

### 3.14. Dissolution Test

For the dissolution tests, the USP apparatus 2, paddle (Erweka DT-D6, Erweka GmbH, Dusseldorf, Germany) was used at 37.0 ± 0.5 °C and 50 rpm (three replicates). In 900 mL of 0.01 N hydrochloric acid pH = 2, which is the medium requested by the USP monograph (Pimozide Tablets, Official Monograph. In The United States Pharmacopeia (USP43-NF38); United States Pharmacopeia Convention, Inc.: Rockville, MD, USA, 2023; p. 3548), it can simulate a fasted stomach environment and, in other two fluids, simulates the following: pH 4.5 phosphate buffer (a condition that simulates the gastric environment in fed conditions) and deionized water (neutral conditions). The dissolution media were prepared according to the reagent and buffer solutions section of the USP [33]. All of the samples contain 4 mg of *PMZ*. The drug concentration was determined using UV detection (Lambda 25, Perkin-Elmer, Monza, Italy), and the data were analyzed using the software provided (Winlab V6 software, Perkin-Elmer, Monza, Italy).

## 4. Conclusions

The goal of synthesizing a new binary crystalline entity between pimozide and adipic acid was successfully achieved. The product was characterized from a physico-chemical point of view, and the single-crystal X-ray diffraction demonstrated that it is a salt of formula *[PMZH]*_2_*[adipate]* obtained from the reaction of *PMZ* and *AAD* in a 0.66:0.33 molar ratio.

Other strong points of this work are as follows: –the development of an experimental design which involved the preparation of systems with *PMZ* molar fraction which covers the entire range of compositions, –and the use of the DSC technique to its full potential, i.e., with a great attention to the qualitative behavior, and the quantitative processing of the thermal data (melting enthalpy).

The thermodynamic model proposed based on the qualitative analysis of the DSC curves was confirmed by a good agreement between the experimental and the expected melting enthalpies. 

The new product showed much improved pharmaceutical properties in terms of solubility and wettability compared to the pure drug, both in deionized water and in the two biorelevant fluids simulating oral administration in fed and fasted conditions. A tablet formulation was therefore designed that can release the entire dose of the drug in a very short time. The tablets containing the new crystalline form can make this virtually insoluble drug available for absorption within minutes regardless of the variability of gastric conditions (fasting or fed stomach). 

## Figures and Tables

**Table 1:** Experimental and expected enthalpy changes for the melting peak of *NC* ($∆H_{Exp,\;NC}$ and $∆H_{Th,\;NC}$) and excess *PMZ* ($∆H_{Exp,PMZ}$ and $∆H_{Th,\;PMZ})$.

Sample	$∆H_{Th,NC}$ (J g^−1^)	$∆H_{Exp,NC}$ (J g^−1^)	$∆H_{Th,PMZ}$ (J g^−1^)	$∆H_{Exp,PMZ}$ (J g^−1^)
*PMZ:AAD* 0.9:0.1	26.8	25.7 ± 1.1	94.7	94.5 ± 3.2
*PMZ:AAD* 0.8:0.2	57.8	55.9 ± 0.7	83.2	81.0 ± 0.4
*PMZ:AAD* 0.7:0.3	94.1	94.7 ± 4.1	67.5	70.8 ± 6.5

**Table 2:** Experimental and expected enthalpy changes for the melting peak of *EU* and *AAD* ($∆H_{Exp,\;TOT}$ and $∆H_{Th,\;TOT})$.

Sample	$∆H_{Th,TOT}$ (J g^−1^)	$∆H_{Exp,TOT}$ (J g^−1^)
*PMZ:AAD* 0.2:0.8	122.6	131.0 ± 11.2
*PMZ:AAD* 0.1:0.9	175.5	184.1 ± 9.2

**Table 3:** Experimental and expected enthalpy changes for the melting peak of EU ($∆H_{Ex,\;pEU}$ and $∆H_{Th,\;EU}$) and excess *NC* ($∆H_{Exp,NC}$ and $∆H_{Th,\;NC})$.

Sample	$∆H_{Th,EU}$	$∆H_{Exp,EU}$	$∆H_{Th,NCeccEU}$	$∆H_{Exp,NCeccEU}$	$∆H_{Th,PMZ}$	$∆H_{Exp,PMZ}$
*PMZ:AAD* 0.6:0.4	10.9	7.2 ± 2.9	94.4	94.0 ± 3.3	49.3	50.9 ± 3.6
*PMZ:AAD* 0.5:0.5	30.1	26.6 ± 3.5	70.9	73.7 ± 14.7	27.0	27.1 ± 4.8
*PMZ:AAD* 0.4:0.6	53.8	61 ± 12.4	42.3	44.6 ± 4.4	0.0	0.0

**Table 4:** Assignment of IR peaks/bands to the vibrational modes of the pimozide molecule.

Wavenumbers (cm^−1^)	Vibrations
3121.9	N-H stretching
2956.5, 2935.1, 2852.3, 2805.5	aliphatic C-H stretching
1695.4	C=O stretching
1625.6, 1616.4	N-H bending
1505.5, 1482.2	aromatic ring stretching
1466.2	C-N bending
1372.9	C-O stretching
1153.4	C-F bending
825.4	C-H (1,4-disubstituted ring) out-of-plane bending
661.3	N-C=O bending + N-H bending

**Table 5:** Assignment of IR peaks/bands to the vibrational modes of the adipic acid molecule.

Wavenumbers (cm^−1^)	Vibrations
3500–2500	O-H stretching
2960.9, 2950.5, 2918.1, 2877.0	aliphatic C-H stretching
1684.9	C=O stretching
1461.6	C-H bending

**Table 6:** Solubility of *PMZ* and *PMZ:AAD 0.66:0.33ev* in pH 2.0, pH 4.5, and deionized water at 21 °C.

Solubility at Equilibrium (mg L^−1^) at 21 °C			
Sample	pH 2.0	pH 4.5	H_2_O
*PMZ*	175.4 ± 1.8	6.4 ± 0.2	1.4 ± 0.3
*PMZ:AAD 0.66:0.33ev*	335.1 ± 3.6	27.3 ± 0.5	8.9 ± 0.4

**Table 7:** Single tablet composition of *PMZ:AAD 0.66:0.33tab* formulation corresponding to a dose of 4 mg of drug.

Single-Tablet Composition	
*PMZ:AAD 0.66:0.33ev* (corresponding to 4 mg of *PMZ*)	4.65 mg
Microcrystalline cellulose NF	115.0 mg
Sodium starch glycolate (Explotab)	35.0 mg
Polyoxyethylene polyoxypropylene triblock copolymer (Poloxamer 407)	20.0 mg
Sodium laurylsulfate	5.0 mg
Magnesium stearate	1.0 mg
Silicon dioxide (Syloid 72FP)	1.0 mg
Total Weight	181.65 mg

## Data Availability

The data presented in this study are available on request from the corresponding author.

## Supplementary Materials

The following supporting information can be downloaded at: https://www.mdpi.com/article/10.3390/molecules29235610/s1, Figure S1: Tammann’s plot for the melting peak of NC; Figure S2: TGA curve of the *PMZ:AAD* 0.66:0.33 sample; Figure S3: Comparison between the experimental PXRD pattern for the *PMZ*:*AAD* 0.67:0.33 composition and the calculated one from single-crystal data at room temperature; Figure S4: Comparison between the experimental PXRD pattern for the *PMZ*:*AAD* 0.7:0.3 composition and the calculated one from single-crystal data at room temperature, the calculated one for adipic acid, the experimental one for pimozide; Figure S5: Comparison between the experimental PXRD pattern for the *PMZ*:*AAD* 0.5:0.5 composition and the calculated one from single-crystal data at room temperature, the calculated one for adipic acid, the experimental one for pimozide; Figure S6: Comparison between the experimental PXRD pattern for the *PMZ*:*AAD* 0.3:0.7 composition and the calculated one from single-crystal data at room temperature, the calculated one for adipic acid, the experimental one for pimozide; Figure S7: The pattern of hydrogen bonding distances at 150 K and at RT; Table S1: Crystal data and details of measurement for *[PMZH]*_2_*[adipate]* at RT and 150 K.
