# Supplementary material for: Pimozide and Adipic Acid: A New Multicomponent Crystalline Entity for Improved Pharmaceutical Behavior
Source: Molecules. 2024 Nov 27;29(23):5610. doi: 10.3390/molecules29235610 (PMC11643450; doi:10.3390/molecules29235610)
Supplement: Supplementary file 1 [file molecules-29-05610-s001.zip › molecules-3301890-supplementary.pdf]

Supplementary material for:

Pimozide and adipic acid: a new multicomponent crystalline entity for improved pharmaceutical behavior

Alessandra Buscarini [1], Michael J. Zaworotko [2], Catiúcia R. M. O. Matos [2], Fabrizia Grepioni [3], Laura Contini [3], Doretta Capsoni [1], Valeria Friuli [4], Lauretta Maggi [4], Giovanna Bruni [1,*]

[1] Department of Chemistry, Physical Chemistry Section & C.S.G.I. (Consorzio Interuniversitario per lo Sviluppo dei Sistemi a Grande Interfase), University of Pavia, via Taramelli 16, 27100 Pavia, Italy; giovanna.bruni@unipv.it (G.B.); alessandra.buscarini01@universitadipavia.it (A.B.); doretta.capsoni@unipv.it (D.C.)

[2] Department of Chemical Sciences and Bernal Institute, University of Limerick, Limerick V94 T9PX, Ireland; xtal@ul.ie (M.Z.); Catiucia.Matos@ul.ie (C.M.)

[3] Dipartimento di Chimica "Giacomo Ciamician", Università di Bologna, Via Selmi 2, 40126 Bologna, Italy; fabrizia.grepioni@unibo.it (F.G.); laura.contini3@unibo.it (L.C.)

[4] Department of Drug Sciences, University of Pavia, viale Taramelli 12, 27100 Pavia, Italy; lauretta.maggi@unipv.it (L.M.) valeria.friuli@unipv.it (V.F.).

* Correspondence: giovanna.bruni@unipv.it

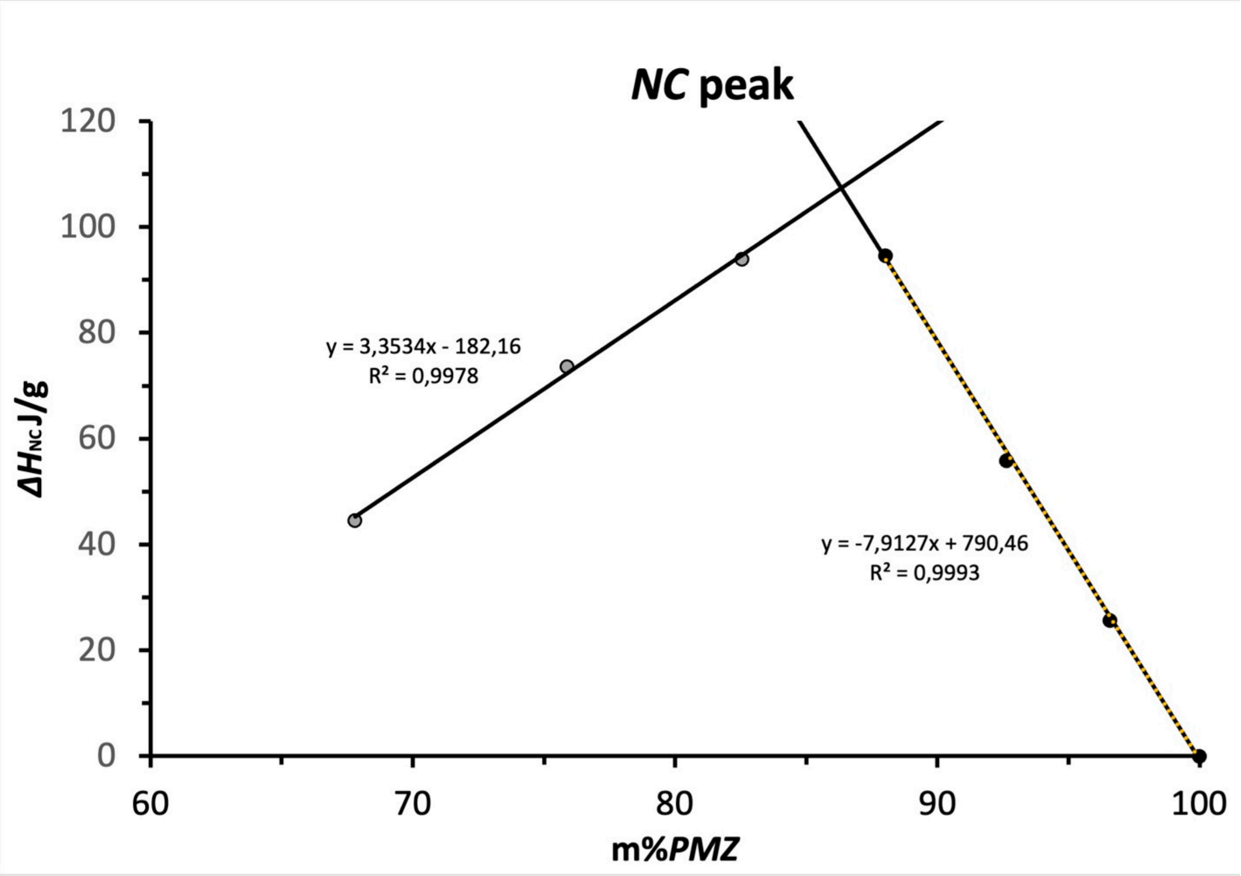

Figure S1. Tammann's plot for the melting peak of *NC*.

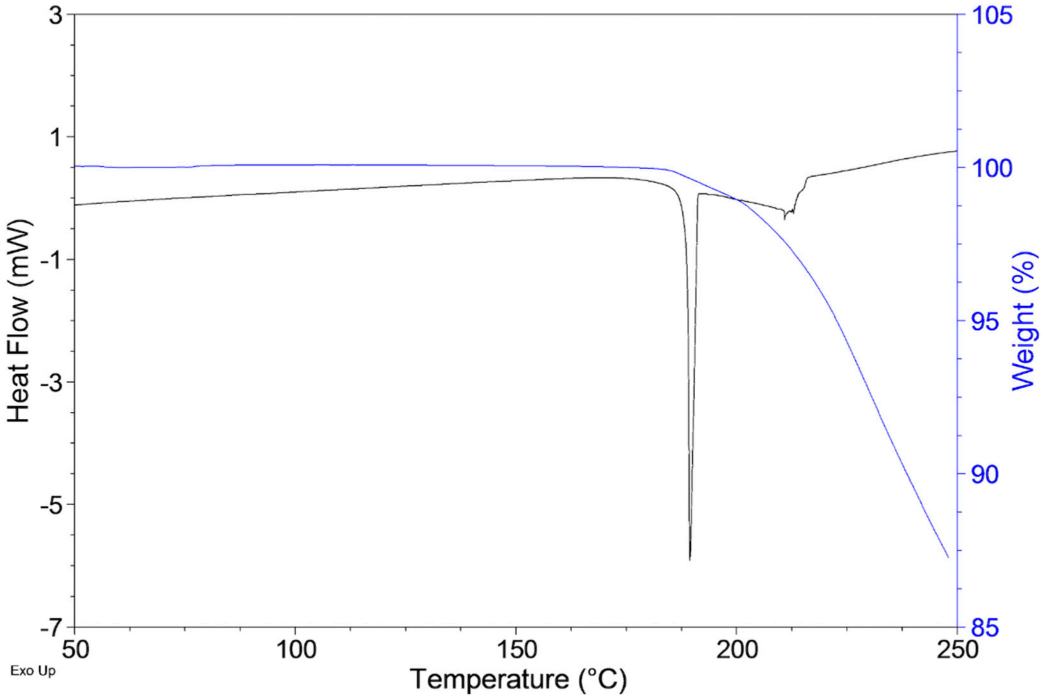

Figure S2. TGA curve of the *PMZ:AAD* 0.66:0.33 sample.

Table S1: Crystallographic data and details of measurement for $[PMZH]_2$[adipate] at RT and 150 K.

	**$[PMZH]_2$[adipate] RT**	**$[PMZH]_2$[adipate] 150 K**
Chemical formula	$C_{31}H_{34}N_3O_3F_2$	$C_{31}H_{34}N_3O_3F_2$
Formula weight	534.61	534.61
Temperature /K	298	150
Crystal system	monoclinic	monoclinic
Space group	$P2_1/n$	$P2_1/n$
a /Å	11.8567(4)	11.7664(3)
b /Å	18.9205(7)	18.7447(5)
c /Å	12.1859(4)	12.0984(3)
α /°	90	90
β /°	96.623(3)	96.4220(10)
γ /°	90	90
Volume /Å^3	2715.48(16)	2651.65(12)
Z, Z’	4, 1	4, 1
d_{calc} / g cm^{-3}	1.308	1.339
μ / mm^{-1}	0.094	0.096
Refls. measd./unique	12739/6286	67167/6610
R_{int}	0.0254	0.0345
Goodness-of-fit on F^2	1.098	1.096
R_1 [I >2σ(I)]	0.0810	0.0661
wR_2 [all data]	0.1763	0.1442

CCDC 2389843-2389844 contain the supplementary crystallographic data for this paper. These data can be obtained free of charge via www.ccdc.cam.ac.uk/data_request/cif, or by emailing data_request@ccdc.cam.ac.uk, or by contacting The Cambridge Crystallographic Data Centre, 12 Union Road, Cambridge CB2 1EZ, UK; fax: +44 1223 336033.

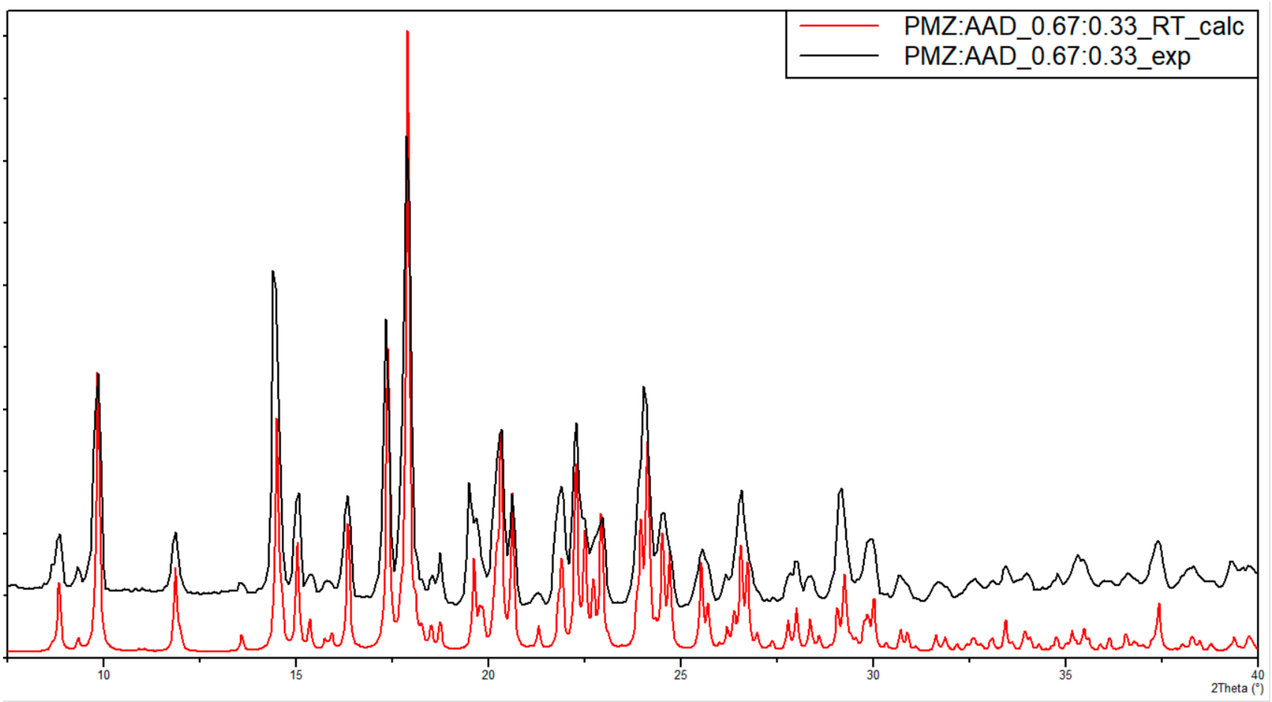

Figure S3. Comparison between the experimental PXRD pattern for the *PMZ:AAD* 0.67:0.33 composition (black line) and the calculated one from single crystal data at room temperature (red line).

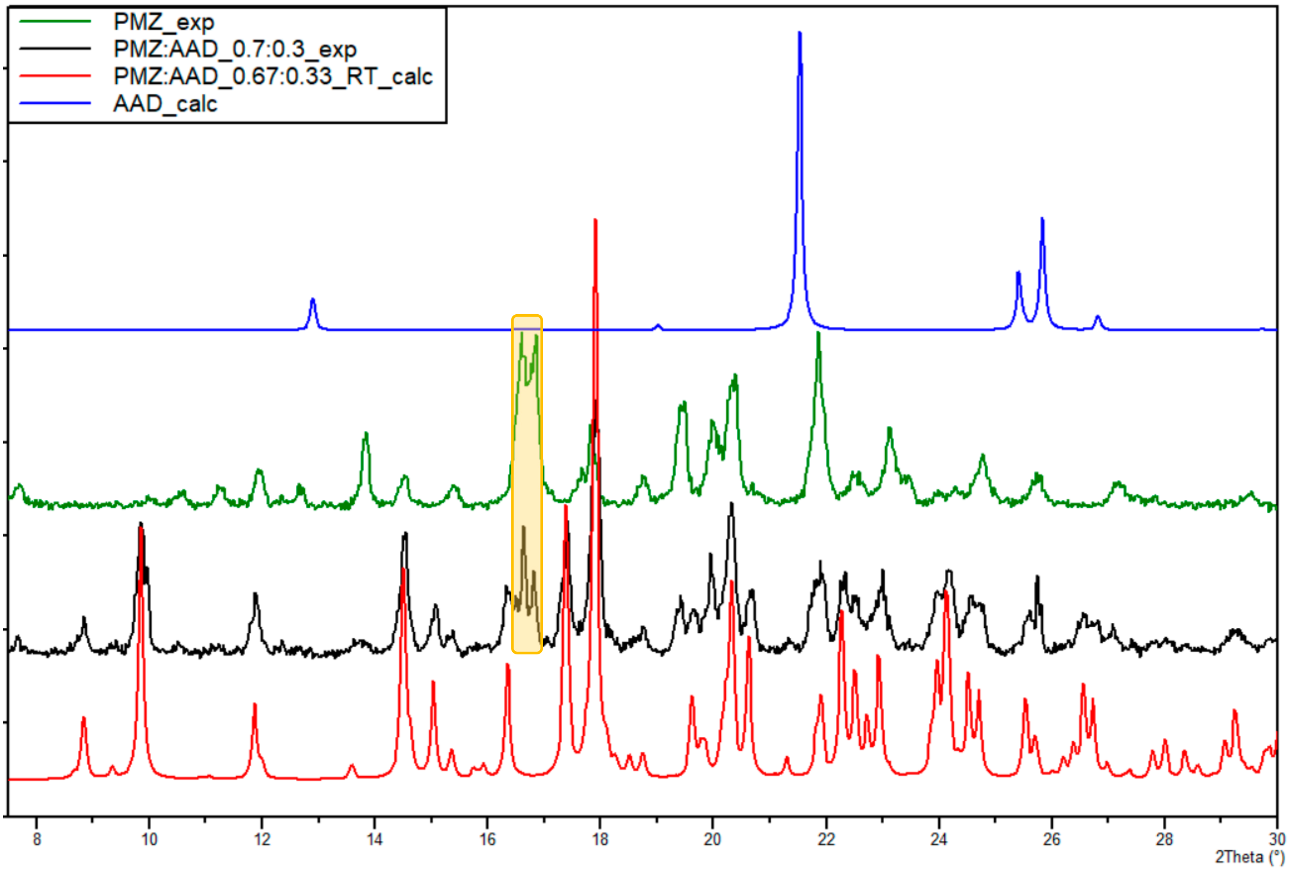

Figure S4. Comparison between the experimental PXRD pattern for the *PMZ:AAD* 0.7:03 composition (black line) and the calculated one from single crystal data at room temperature(red line), the calculated one for adipic acid (blue line), the experimental one for pimozide (green line). The yellow frame evidences the presence of unreacted pimozide in the 0.7:0.3 composition.

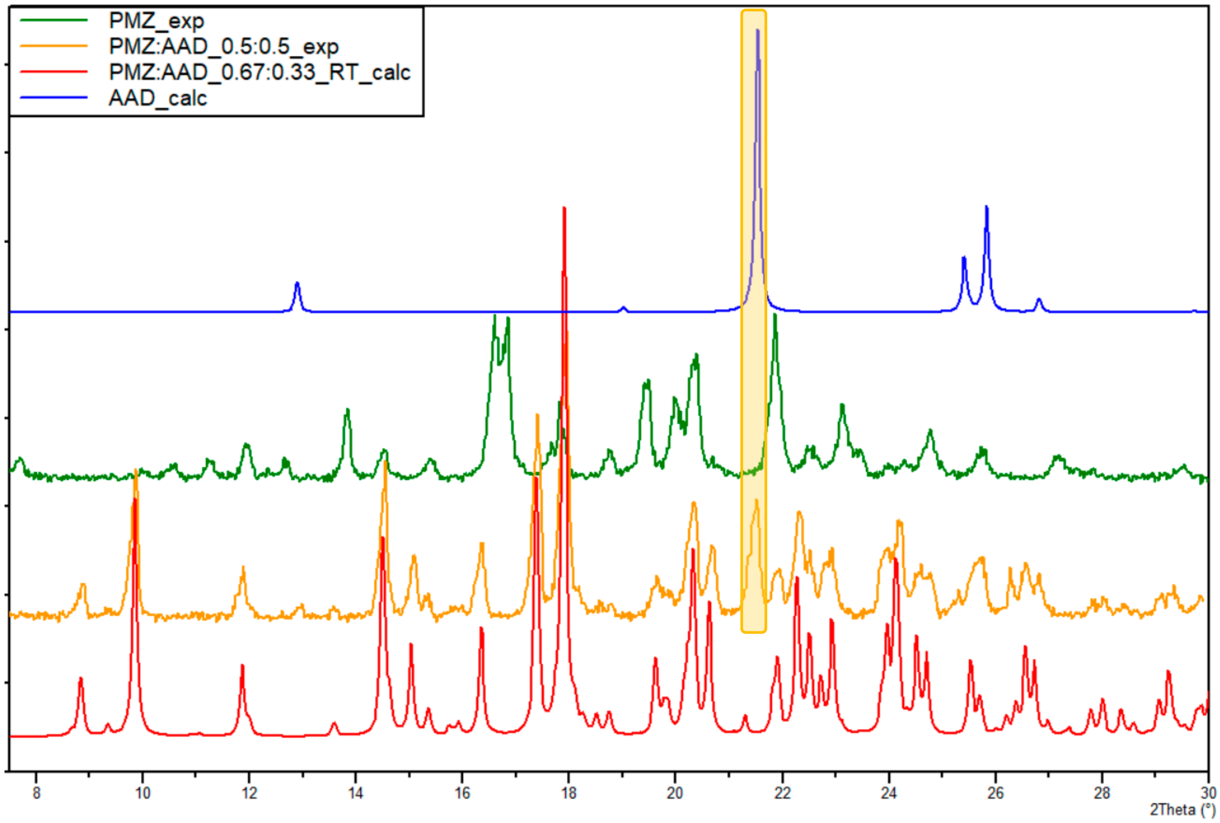

Figure S5. Comparison between the experimental PXRD pattern for the *PMZ:AAD* 0.5:0.5 composition (yellow line) and the calculated one from single crystal data at room temperature(red line), the calculated one for adipic acid (blue line), the experimental one for pimozide (green line). The yellow frame evidences the presence of unreacted adipic acid in the 0.5:0.5 composition.

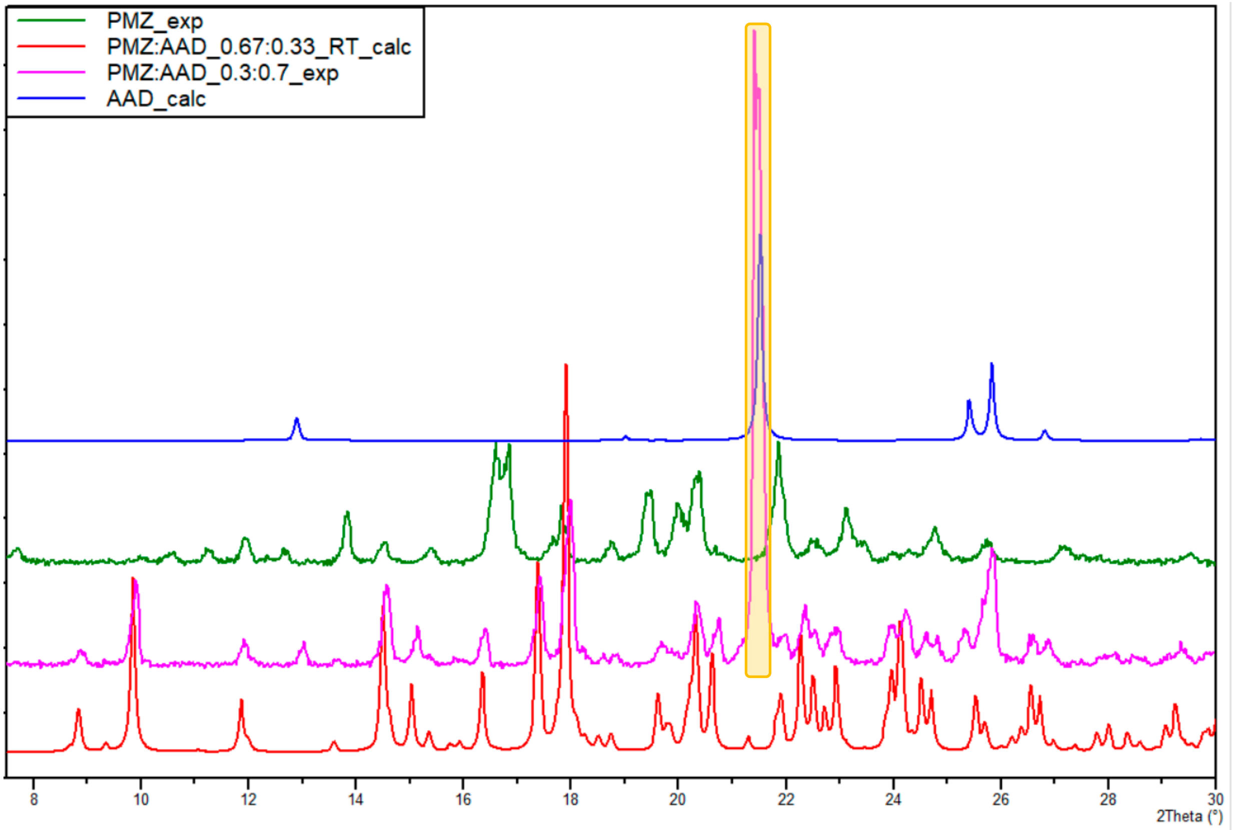

Figure S6. Comparison between the experimental PXRD pattern for the *PMZ:AAD* 0.3:0.7 composition (yellow line) and the calculated one from single crystal data at room temperature(red line), the calculated one for adipic acid (blue line), the experimental one for pimozide (green line). The yellow frame evidences the presence of a large amount of unreacted adipic acid in the 0.3:0.7 composition.

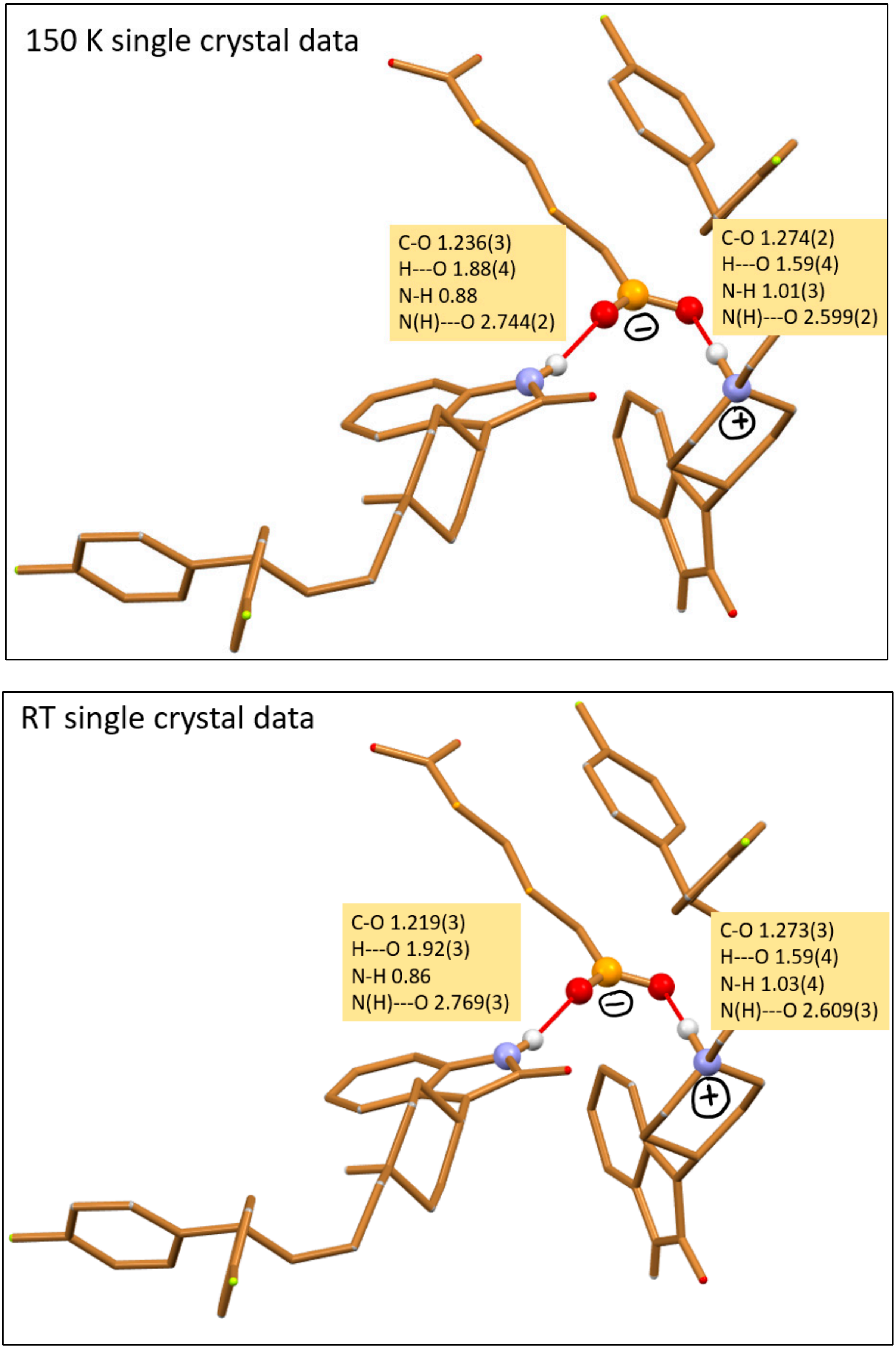

Figure S7. The pattern of hydrogen bonding distances at 150 K and at RT is in agreement with the formation of a salt for the *PMZ*:*AAD* 0.66:0.33 composition: (i) the values of the C-O bond distances are too close to each other to be an indication of the presence of neutral carboxylic group; (ii) the N(H)···O distances are significantly shorter when they involve the protonated nitrogen, i.e., when the hydrogen bond is charge-assisted both via the donor and the acceptor atoms.
